# The Relationship Between Women’s Negative Body Image and Disordered Eating Behaviors During the COVID-19 Pandemic: A Cross-Sectional Study

**DOI:** 10.3389/fpsyg.2022.856933

**Published:** 2022-03-24

**Authors:** Giulia Corno, Amélia Paquette, Johana Monthuy-Blanc, Marilou Ouellet, Stéphane Bouchard

**Affiliations:** ^1^Laboratory of Cyberpsychology, Department of Psychology and Psychoeducation, Université du Québec en Outaouais, Gatineau, QC, Canada; ^2^GR2TCA-Loricorps-Groupe de Recherche Transdisciplinaire des Troubles du Comportement Alimentaire, Université du Québec à Trois-Rivières, Trois-Rivières, QC, Canada; ^3^Centre de Recherche de l’Institut Universitaire en Santé Mentale de Montréal, Montréal, QC, Canada

**Keywords:** body dissatisfaction, weight concerns, shape concerns, negative body image, disordered eating, COVID-19, women’s health

## Abstract

Recent studies have shed light on how the COVID-19 pandemic changed our lives, and most of them have documented its detrimental effect on eating habits. Until now, the effects of this global crisis on negative body image and its association with disordered eating behaviors remain largely understudied. This study aimed to investigate changes in frequency of disordered eating behaviors (i.e., restrictive eating, emotional eating, and overeating) and negative body image (i.e., shape and weight concern, and body dissatisfaction) among a community sample of women during the COVID-19 pandemic (October 2020–May 2021). Furthermore, we explored the possible relation between body image-related variables and changes in the frequency of disordered eating behaviors in the context of the pandemic. A total of 161 self-identified female participants enrolled in an online-based survey. Descriptive statistics showed that women did not report clinically significant levels of weight and body shape concerns, but participants reported being dissatisfied with their body. One sample Wilcoxon Signed Rank tests revealed a tendency toward an increasing of the frequency of all disordered eating behaviors during the COVID-19 pandemic. Multinomial logistic regressions showed that weight concerns predicted an overall increase in the frequency of restrictive eating behaviors, whereas higher body dissatisfaction was associated with a moderate self-perceived increase in the frequency of emotional eating. These results shed light on a risk pattern of phenomena in a non-clinical sample of women, as they represent the key risk factors for the development of eating disorders. Findings could have implications for designing and implementing prevention programs.

## Introduction

In December 2019, the world became aware of the development of a novel coronavirus, the coronavirus disease 2019 (COVID-19, World Health Organization, 2020a, 2020b). To limit the spread of COVID-19, a high proportion of countries implemented severe restrictions on social life, travels, and lockdown periods. While these measures were essential to limit the disease spread, there is increasing evidence of negative impact of this pandemic and lockdown responses on mental health of the general population, with increased levels of anxiety, stress, depression, emotional exhaustion, negative social emotions (e.g., shame and guilt), sleeping issues, feelings of loneliness, and cases of post-traumatic stress and suicide (Brooks et al., 2020; Cavalera, 2020; de Medeiros Carvalho et al., 2020; Galea et al., 2020; McCombie et al., 2020; Torales et al., 2020; Ramalho et al., 2021).

Pandemic-related restrictions and lockdown periods significantly changed daily routines. Home confinement and food insecurity could have an impact on food consumption and eating habits. On one hand, they could increase restrictive eating (e.g., restrictive calories intake and skipping meals), but on the other hand, they may also foster overeating and binge eating episodes as consequences of the increased availability of food at home brought and stored up in response to general food insecurity and increased time spent at home (Scarmozzino and Visioli, 2020; Schlegl et al., 2020; Touyz et al., 2020; Weissman et al., 2020). Furthermore, boredom from being forced to stay at home could have promoted overeating as a way to escape the monotony, whereas negative experiences could give rise to restrictive eating patterns due to a stress reaction that emulate the interoceptive sensations associated to satiety (Crockett et al., 2015; Sanlier et al., 2021). By the same token, the pandemic-related stress could have led to emotional eating as a mechanism to cope with mood changes and negative emotions (e.g., shame and guilt; Cavalera et al., 2018; Ferrell et al., 2020; Madali et al., 2021; Sanlier et al., 2021).

Research conducted so far points to a marked increase of irregular eating patterns since the beginning of the pandemic (Flaudias et al., 2020, 2021; Ismail et al., 2020). In line with these studies, an international online survey demonstrates the negative effects of the pandemic on dietary habits, such as increasing unhealthy food consumption, overeating, meal skipping, snacking, and loss of control (Ammar et al., 2020). A large-scale survey including an Australian community and clinical sample (Phillipou et al., 2020) reports that, since the beginning of the pandemic, 27.6% of their community sample reported to have increased the frequency of restrictive eating behaviors as a way to control their shape and weight and 34.6% reported increased binge eating behaviors, despite any antecedent of eating disorders. Ramalho et al. (2021) denounce a variousness of disordered eating behaviors as skipping meals (52.8%), overeating (81%), binge eating episodes (39.2%), grazing eating behavior (80.9%), and loss of control over eating (47.2%) among a Portuguese community population 1 week after the end of the first lockdown period (i.e., May 11th, 2020). Results from an Italian large-scale survey show that most of the participants reported a change in their hunger and satiety perception as well as in their dietary habits (Di Renzo et al., 2020). Notably, women were more likely to report increasing struggles with regulating eating and preoccupation with food (Robertson et al., 2021).

There is also preliminary evidence of increased concerns about appearance and heightened weight and shape concerns in people from the United Kingdom’s general population during the COVID-19 pandemic (Robertson et al., 2021). Several studies suggest strong links between exposure to media and social network harmful appearance-related content and disordered eating attitudes/behaviors (e.g., Groesz et al., 2002; Agliata and Tantleff-Dunn, 2004; Glauert et al., 2009; Hawkins et al., 2010; Marques et al., 2022). In the context of the COVID-19 pandemic, evidence shows that the lack of access to in person social interactions due to confinement has led to an increase in media consumption and social networking, exposing confined individuals to a more thin/athletic ideal (Cooper et al., 2020). Increased consumption of social media and daily screen-time during the lockdown, collective concerns about weight gain, and messages conveyed by social media about the dangers of being overweight might significantly influence one’s body image concerns (Flaudias et al., 2020; Vall-Roqué et al., 2021). COVID-19-related stress and anxiety are also found to be associated with more negative body image (Swami et al., 2021). Indeed, Swami et al. (2021) propose a pathway linking COVID-19 stress/anxiety to a worsening of negative body image: COVID-19-related stress and anxiety could undermine one’s coping resources to face increasing threats to body image, such as an increased exposure to thin/athletic bodies (e.g., due to the increased usage of social media since the pandemic onset; see Cooper et al., 2020; Koeze and Popper, 2020; Pietrobelli et al., 2020), greater worries about body weight and shape (e.g., due to exercise limitations especially during lockdown, see Cooper et al., 2020; Mattioli et al., 2020), and increased frequency of negative body rumination.

Negative body image has been found to be related to disordered eating behaviors (e.g., Stice et al., 1996, 1998; Cash and Deagle, 1997; Burrows and Cooper, 2002; Neumark-Sztainer et al., 2003; Levine and Piran, 2004; Andersen and Swami, 2021) and to be a well-established risk factor for the development and maintenance of eating disorders (e.g., Stice, 2001, 2002; Fairburn et al., 2003; Glashouwer et al., 2019). Nevertheless, the impact of COVID-19 crisis on body image issues and their association with disordered eating behaviors remains largely understudied. Understanding how the COVID-19 pandemic affected body image and problematic eating habits is of critical importance as the adverse consequences related to the pandemic could result in greater risk of development of eating disorders and the aggravation of symptoms in individuals already suffering from an eating disorder. Indeed, Rodgers et al. (2020) identify three different pathways that could promote the development or enhancement of eating disorder symptomatology during the COVID-19 pandemic. The first pathway suggests that restrictions placed on people’s movements, daily routine, and outdoor activities might increase weight and shape concerns as well as eating, exercising, and sleeping patterns. The second pathway proposes that media consumption related to the COVID-19 pandemic (i.e., exposure to harmful eating and appearance-related media, increased consumption of global social media, and use of video-conferencing) may increase eating disorders risk and symptoms. The third pathway suggests that the fear of contagion from COVID-19 might augment stress and avoidance of food believed to be impure or to be a vehicle of contagion. These orthorexia-based cognitions could subsequently increase restrictive eating patterns (Rodgers et al., 2020).

In consideration of the aforementioned findings and their possible preventive and therapeutic implications, we investigated self-reported changes in frequency of disordered eating behaviors (i.e., restrictive eating, emotional eating, and overeating) and negative body image (i.e., shape and weight concern and body dissatisfaction) among a community sample of women during the COVID-19 pandemic. Furthermore, we explored the possible relationship between body image-related variables and perceived changes in the frequency of disordered eating behaviors in the context of the COVID-19 pandemic. In light of the literature on problematic eating behaviors during the COVID-19 pandemic, we expected an increase in disordered eating patterns since the beginning of the COVID-19 crisis. Regarding negative body image, since we were interested in a community sample of women, we did not expect clinical levels of body image-related issues. Nevertheless, since negative body image is considered as normative (i.e., “normative discontent,” see Rodin et al., 1984; Tantleff-Dunn et al., 2011; Tiggemann, 2011; Siegel et al., 2020) among women in Western cultures, we expected to find non-clinical levels of shape and weight concerns as well as body dissatisfaction. Given the aforementioned relation between negative body image issues and problematic eating behaviors, we hypothesized a positive relationship between these two phenomena. However, previous data regarding the COVID-19 pandemic period do not exist to guide predictions as to the relative strength of these relationships.

## Materials and Methods

### Participants

The sample consisted of 197 female self-identified participants, who accepted participating in an online-based survey exploring the impact of the COVID-19 pandemic on the self. Inclusion criteria were as: over 18 years old, self-identifying as female, never having been diagnosed with EDs, having access to the Internet, being Canadian residents, and being able to read and understand French. Almost all participants (98.2%) self-reported to be Canadian residents. Participants (*n* = 3) who self-reported to be resident in a country other than Canada were excluded. To provide context for the situation during the recruitment period, Canada had reached a critical level of COVID-19 cases in the population. Between October 2020 and December 2020, most regions went into maximum alert, which involved closing non-essential businesses, restricting inter-regional travel, and banning gatherings, a curfew in effect from 8 p.m. to 5 a.m., mandatory teleworking and online school. Canada experienced several tightening of regulations until May 2021, with a slight loosening in March 2021 due to the start of vaccination (e.g., Gouvernement du Québec, 2021). Data collection was ended when most of the pandemic-related restrictions started to be removed in order to preserve data from potential bias. Finally, individuals (*n* = 33) with a self-reported current or past diagnosed eating disorder were excluded from this study, yielding a sample size of *n* = 161.

### Procedure

Recruitment took place over seven (7) months, from October 2020 to May 2021. The study was advertised on social media and participants were recruited using opportunistic and snowball sampling. Participants completed an anonymous online-based survey on LimeSurvey containing sociodemographic questions and questions around their eating habits and body image during the pandemic. Informed consent was obtained prior to completing the survey. The present study was carried out in compliance with current legislation regarding the protection of personal data (Helsinki Declaration of 1975, as revised in 2018, and the Tri-Council Policy Statement: Ethical Conduct for Research Involving Humans—TCPS 2 of 2018) and obtained the approval from the ethical committee of Université du Québec en Outaouais (Quebec, Canada) and of Université du Québec à Trois-Rivières (Quebec, Canada).

### Measures

#### Demographic Information

Participants were asked information about self-reported age, gender, country of residency, relationship status, level of education, skin color, and self-reported current height and weight (to calculate Body Mass Index, BMI). In addition, participants were asked if they ever received (in their past or present) an eating disorder diagnosis. In the present study, participants were considered to have never received a diagnosis of an eating disorder if they answered no to either question.

#### Self-Rated Changes in the Frequency of Disordered Eating Behaviors

Participants were asked, “Since the COVID-19 pandemic, please rate each of the following using this scale:,” ranging from “1 = Much less than before,” “2 = Less than before,” “3 = Somewhat less than before,” “4 = No change than before,” “5 = Somewhat more than before,” “6 = More than before,” and “7 = Much more than before.” Items included “Emotional eating (overeating in response to negative emotions such as anxiety or irritability)” (Van Strien et al., 2007), “Restrictive eating (deliberately trying to limit the amount of food you eat or exclude any food from your diet to influence your shape or weight),” and “Overeating (eating an unusually large amount of food given the circumstances)” (Fairburn and Cooper, 1993; Fairburn and Beglin, 1994).

#### Shape Concerns and Weight Concerns

Participants were asked to answer the body shape and weight concerns subscales of the Eating Disorder Examination Questionnaire (EDE-Q; Fairburn and Cooper, 1993; Fairburn and Beglin, 1994). The EDE-Q is a 28-items questionnaire derived from the Eating Disorder Examination (EDE; Cooper and Fairburn, 1987; Fairburn and Cooper, 1993) interview. It is a well-established self-report questionnaire measuring eating disorder-related attitudes and behaviors and is used for research among community and clinical samples. Items of the EDE-Q are scored on a 7-point scale. Subscale and global scores indicate the severity of eating disorder psychopathology. Scores of 4 or higher indicate a clinical range (Carter et al., 2001). In the present study, the EDE-Q showed good internal consistency for both the subscales (Shape concern: Cronbach’s *α* = 0.905, McDonald’s *ω* = 0.906; Weight concern: Cronbach’s *α* = 0.808; and McDonald’s *ω* = 0.825).

#### Body Dissatisfaction

**Figure 1:**
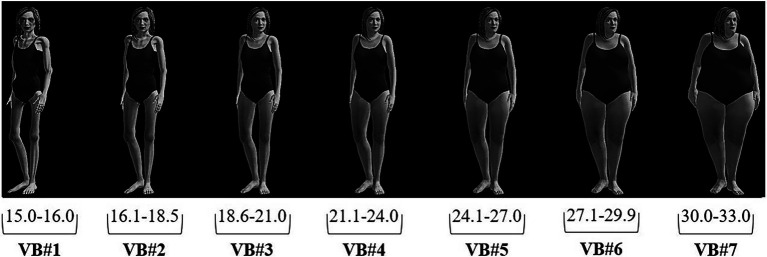
The *e*LoriCorps-IBRS 1.0.

Body dissatisfaction was calculated using the *e*LoriCorps Immersive Body Rating Scale (*e*LoriCorps-IBRS 1.0; Monthuy-Blanc et al., 2020). This scale is composed of seven virtual bodies of increasing BMIs, from 15 to 33 kg/m^2^ (Figure 1). Since the scale was available only with light skin color virtual bodies, in the present study, virtual bodies were presented in shadows of grey in order to be more inclusive. However, the virtual bodies did not represent the morphological characteristics of women with dark or very dark skin. To control the potential lack of representativeness, we asked participants to self-report their skin color as mentioned above. This scale was used in order to perform visual depictive body size estimation tasks (Moelbert et al., 2017). Participants were asked to choose between a line-up of seven 3D bodies (presented in a third person-allocentric perspective), the one that better represents their perceived body, and the one that better represents their ideal body. Body dissatisfaction corresponds to the perceived body size minus the ideal body size. A positive score indicates that a participant’s ideal body is thinner than their perceived body, whereas a negative score indicates that a participant’s ideal size is bigger than their perceived size. A score of 0 indicates that there is no difference between ideal and perceived body size (i.e., participants are not dissatisfied with their body). Body dissatisfaction scores can range from −6 to +6.

### Data Analysis Plan

Descriptive statistics were used to paint a portrait about body image-related variables and self-perceived changes in the frequency of disordered eating habits (i.e., restrictive eating, overeating, and emotional eating) since the COVID-19 outbreak. One sample Wilcoxon Signed Rank tests were used to compare whether sample means for each eating habit differed from a null hypothesis of “No change than before” (corresponding to a rating of 4 in the questionnaire developed for this study aims). One sample *t*-tests were run to further probe these findings. Non-parametric correlation analysis was used to explore the relationship between the three disordered eating habits and body image-related measures. Because ratings on these items represented an ordinal versus continuous variable, we conducted a second set of parametric correlation analysis. Finally, we conducted multinomial (polychotomous) logistic regressions in order to predict several categorical outcomes (changes in the frequency of each disordered eating habit) from continuous predictors (body image-related variables). Cohen’s *d* effect sizes and Nagelkerkes’s *R*^2^ coefficients of determination are reported (Cohen, 1988; Field, 2018). The z-scores of Kurtosis and Skewness of the continuous variables were analyzed to verify their normal distribution (at *p* < 0.05; Field, 2018). Pairwise deletion was used to handle missing data and statistics were performed using IBM SPSS, version 27.

## Results

Mean participants age was 35.72 (SD = 13.87). Self-reported mean height was 162.91 cm (SD = 5.85), and weight was 72.89 kg (SD = 19.62), resulting in an average BMI of 27.41 kg/m^2^ (SD = 6.97). Three (3) participants reported that their level of education was less than high school (1.9%), 101 participants obtained a high school diploma (62.7%), 8 participants obtained a university certificate (5%), and 25 a bachelor’s degree (15.5%), whereas 24 reported a postgraduate level of education (14.9%). Regarding self-reported relationship status, 87 participants reported being married or in a common-law relationship (54.1%), 11 participants reported being divorced or separated (6.8%), 29 to be in a relationship (18%), 33 did not have a partner (20.5%), and 1 person preferred not to answer (0.6%). Concerning self-reported skin color, 38 participants self-identified their skin color as very light (38.8%), 43 as light (43.9%), 14 as intermediate (14.3%), and 3 as tan (3.1%). No participant reported having dark or very dark skin. The skin color scale used in this study is available in Supplementary Material.

### Negative Body Image-Related Variables

Women did not report clinically significant levels of weight (M = 2.18, SD = 1.49, range: 0.00–5.60) and body shape (M = 2.28, SD = 1.54, range: 0.00–5.75) concerns. On average participants reported being dissatisfied with their body (M = 1.01, SD = 0.90, range: −1.00-3.00), desiring a thinner body (M = 3.48, SD = 0.82, range: 2.00–6.00) than their perceived one (M = 4.49, SD = 1.08, range: 3.00–7.00). Since no participant self-reported to have dark or very dark skin, we did not conduct any analyses in order to explore the possible effect of the lack of representativeness of the virtual bodies.

### Self-Perceived Changes in the Frequency of Eating Habits

Table 1 presents participants’ self-reported changes since the COVID-19 outbreak in frequency of emotional eating, overeating, and restrictive eating. Women’s mean scores were significantly different from a rating of “4″ (i.e., no change than before) on all variables (restrictive eating: *p* = 0.012; overeating: *p* = 0.002; and emotional eating: *p* < 0.001). Effect sizes were small for restrictive eating and overeating, and moderate for emotional eating. Differences indicate a tendency to have increased the frequency of restrictive eating, overeating, and emotional eating. Results from parametric analyses paralleled the ones obtained with the non-parametric analyses, indicating that the median value for each eating habit was significantly different than “4” (i.e., no change than before; see Supplementary Material).

**Table 1:** Self-rated changes in frequency of disordered eating behaviors based on retrospective self-report (*n* = 152).

	Restrictive eating	Overeating	Emotional eating
	*n* (%)	*n* (%)	*n* (%)
1 = Much less than before	5 (3.3)	5 (3.3)	2 (1.3)
2 = Less than before	6 (3.9)	3 (2.0)	1 (0.7)
3 = A little less than before	10 (6.6)	4 (2.6)	10 (6.6)
4 = No change from before	80 (52.6)	92 (60.5)	66 (43.4)
5 = A little more than before	33 (21.7)	37 (24.3)	53 (34.9)
6 = More than before	14 (9.2)	7 (4.6)	11 (7.2)
7 = Much more than before	4 (2.6)	4 (2.6)	9 (5.9)
*z*	2.50	3.16	5.98
*r*	0.20	0.26	0.49
Value of p	0.012	0.002	< 0.001

### Self-Reported Changes in the Frequency of Restrictive Eating and Their Relationship With Body Image

Non-parametric correlation analysis between eating habits and body image-related variables are reported in Table 2. Pearson’s correlations are available in Supplementary Material. Self-perceived changes in the frequency of restrictive eating were significantly positively correlated to both body shape (*p =* 0.001) and weight (*p* < 0.001) concerns. A multinomial logistic regression was performed to create a model of the relationship between the predictor variables (i.e., shape and weight concerns) and six outcome categories (i.e., self-rated changes in the frequency of restrictive eating behaviors: “1 = Much less than before,” “2 = Less than before,” “3 = Somewhat less than before,” “5 = Somewhat more than before,” “6 = More than before,” and “7 = Much more than before”) when compared to a baseline outcome category (i.e., “4 = No change than before”). Analysis of collinearity statistics shows the assumption of no multicollinearity had been met since tolerance values were above 0.1 and Variance Inflated Factor (VIF) values were less than 10 (VIF = 4.67, tolerance score = 0.214; Bowerman and O’Connell, 1990; Myers, 1990; Field, 2018, p. 402). Table 3 presents the results of this multinomial logistic regression. The fit between the model containing only the intercept and data significantly improved with the addition of the predictor variables, *χ*^2^(12) = 52.07, *p* < 0.001, Nagelkerkes’s *R*^2^ = 0.351. Pearson Chi-Square test indicated that the model was a good fit to the data (*p* = 0.975). Weight concerns had a significant main effect on self-perceived changes in the frequency of restrictive eating, *χ*^2^(6) = 17.28, *p* = 0.008, whereas shape concerns did not have a significant main effect, *χ*^2^(6) = 4.30, *p* = 0.637. Once the overall effects were broken down, results showed that higher weight concerns significantly predicted a slight (“A little more than before”) *b* = 1.36, Wald *χ*^2^(1) = 12.11, *p* = 0.001, and moderate (“More than before”) *b* = 1.35, Wald *χ*^2^(1) = 6.44, *p* = 0.011 increase in the frequency of restrictive eating behavior. In other words, women were more likely to self-perceive an increase in the frequency of restrictive eating behavior—than not change the frequency—if they experienced higher weight concerns during the COVID-19 pandemic.

**Table 2:** Spearman’s correlation analyses between disordered eating behaviors and negative body image (*n* = 152).

Variable	1	2	3	4	5	6
1. Restrictive eating	–	−0.02	0.06	0.33^***^	0.28^**^	0.15
2. Overeating	−0.02	–	0.41^***^	0.05	0.07	0.19
3. Emotional eating	0.06	0.41^***^	–	0.00	0.00	0.22^*^
4. Weight concerns ^a^	0.33^***^	0.05	0.00	–	0.88^***^	0.51^***^
5. Shape concerns ^b^	0.28^**^	0.07	0.00	0.88^***^	–	0.49^***^
6. Body dissatisfaction ^c^	0.15	0.19	0.22^*^	0.51^***^	0.49^***^	–

^*^p < 0.05; ^**^p < 0.01; and ^***^p < 0.001. N = 152, except for ^a^n = 130, ^b^n = 131, and ^c^n = 97.

**Table 3:** Multinomial logistic regression: weight and shape concerns and their relations with self-rated changes on restrictive eating (*n* = 130).

	*b* (*SE*)	95% CI for Odds Ratio		
		Lower	Odds Ratio	Upper
Much less than before vs. No change than before				
Intercept	−4.39 (1.11)^***^			
Weight concerns	0.71 (0.71)	0.51	2.04	8.21
Shape concerns	0.11 (0.65)	0.31	1.11	3.98
Less than before vs. No change than before				
Intercept	−3.74 (0.97)^***^			
Weight concerns	0.15 (0.74)	0.27	1.16	4.92
Shape concerns	0.39 (0.67)	0.40	1.47	5.49
Somewhat less than before vs. No change than before				
Intercept	−3.14 (0.75)^***^			
Weight concerns	0.96 (0.53)	0.93	2.62	7.44
Shape concerns	−0.39 (0.50)	0.26	0.68	1.80
Somewhat more than before vs. No change than before				
Intercept	−3.00 (0.60)^***^			
Weight concerns	1.36 (0.39)^**^	1.81	3.88	8.33
Shape concerns	−0.44 (0.35)	0.32	0.64	1.28
More than before vs. No change than before				
Intercept	−5.13 (1.03)^***^			
Weight concerns	1.35 (0.53)^*^	1.36	3.86	10.96
Shape concerns	−0.02 (0.47)	0.39	0.98	2.44
Much more than before vs. No change than before				
Intercept	−7.86 (2.37)^**^			
Weight concerns	0.75 (1.04)	0.27	2.11	16.22
Shape concerns	0.87 (1.01)	0.33	2.40	17.31

^*^p < 0.05; ^**^p < 0.01; and ^***^p < 0.001.

### Self-Perceived Changes in the Frequency of Overeating and Their Relationship With Body Image

None of the body image-related variables (i.e., weight and shape concerns and body dissatisfaction) were significantly related to overeating (see Table 2).

### Self-Reported Changes in the Frequency of Emotional Eating and Their Relationship With Body Image

Weight and shape concerns were not significantly correlated to self-rated changes in the frequency of emotional eating, whereas body dissatisfaction was significantly positively correlated to changes in the frequency of emotional eating (*p* = 0.032; see Table 2). A multinomial logistic regression was performed to create a model of the relationship between the predictor variable (i.e., body dissatisfaction) and six outcome categories (i.e., self-perceived changes in the frequency of emotional eating: “1 = Much less than before,” “2 = Less than before,” “3 = Somewhat less than before,” “5 = Somewhat more than before,” “6 = More than before,” and “7 = Much more than before”) when compared to a baseline outcome category (i.e., “4 = No change than before”). The model fit improved with the addition of the predictor variable, *χ*^2^(6) = 18.21, *p* = 0.006, Nagelkerkes’s *R*^2^ = 0.184. Pearson Chi-Square test indicated that the model was a good fit for the data (*p* = 0.604). Once the overall effect of body dissatisfaction was broken down, results showed that higher body dissatisfaction significantly predicted a moderate (“More than before”) increase in the frequency of emotional eating, *b* = 2.25, Wald *χ*^2^(1) = 9.91, *p* = 0.002. Therefore, women were more likely to self-perceive an increase in the frequency of emotional eating—than no change in the frequency—if they experienced higher body dissatisfaction during the COVID-19 pandemic. Table 4 presents the results of this multinomial logistic regression.

**Table 4:** Multinomial logistic regression: body dissatisfaction and its relationship with self-rated changes on emotional eating (*n* = 97).

	*b* (*SE*)	95% CI for odds ratio		
		Lower	Odds ratio	Upper
Much less than before vs. No change from before				
Intercept	−3.30 (1.03)^**^			
Body dissatisfaction	−1.18 (1.30)	0.02	0.31	3.92
Less than before vs. No change from before				
Intercept	−3.30 (1.03)^**^			
Body dissatisfaction	−1.18 (1.30)	0.02	0.31	3.92
Somewhat less than before vs. No change from before				
Intercept	−2.38 (0.69)^**^			
Body dissatisfaction	0.69 (0.49)	0.77	1.99	5.15
Somewhat more than before vs. No change from before				
Intercept	−0.60 (0.36)			
Body dissatisfaction	0.40 (0.29)	0.85	1.49	2.60
More than before vs. No change from before				
Intercept	−5.37 (1.47)^***^			
Body dissatisfaction	2.25 (0.72)^**^	2.34	9.48	38.48
Much more than before vs. No change from before				
Intercept	−3.08 (0.95)^**^			
Body dissatisfaction	0.69 (0.66)	0.55	1.99	7.17

^**^p < 0.01; and ^***^p < 0.001.

## Discussion

To our knowledge, this is the first study which describes self-reported changes in disordered eating behaviors, as well as negative body image among a community population of women during the COVID-19 pandemic. Consistent with our hypotheses, we found a tendency toward a self-rated increase of the frequency of all problematic eating behaviors. We did not find clinical levels of negative body image, but women reported, on average, a certain discontent about their bodies. These results are consistent with the concept of “normative discontent,” which proposes that a negative feeling about one’s physical appearance is thought to be the “norm” in a woman’s life, instead of to be an exception (Rodin et al., 1984; Tantleff-Dunn et al., 2011; Tiggemann, 2011).

Our results showed a significant relation between weight concerns and self-perceived changes in the frequency of restrictive eating during the COVID-19 pandemic. In particular, we found that weight concerns predicted an overall increase in the frequency of restrictive eating behavior. The COVID-19 has brought limitations on physical activities and an increase in time spent indoors (Constandt et al., 2020; Hu et al., 2020). The increase in the rate of depression since the start of COVID-19 (Ettman et al., 2020; Huang and Zhao, 2020; Mazza et al., 2020) may contribute to partly explain the decrease in the level of physical activity and the development of an inactive lifestyle, as these phenomena have been previously associated with each other (Stavrakakis et al., 2012). The aforementioned limitations may as well heighten shape and weight concerns and lead to increased restrictive eating, as theorized by Rodgers and colleagues (Rodgers et al., 2020). Researchers suggest that individuals who follow a restrictive diet composed of multiple requesting rigid food rules to help achieve their ideal body are mainly motivated by high weight and shape concerns (Stice et al., 1996). Stice (2001) proposes a risk factor model in which weight and shape concerns result from an internalization of appearance ideals that leads to eating disorders behaviors *via* dietary restraint and affect regulation mechanisms. In conclusion, the limitations imposed by COVID-19 could have increased concerns about body image and reinforced ideas about ideal appearance, which could possibly have led to increased frequency of restrictive eating.

We found that higher body dissatisfaction was associated with a moderate self-perceived increase in the frequency of emotional eating in our sample during the pandemic. This relation is in line with findings from before the pandemic outbreak (Van Strien et al., 2005; Anschutz et al., 2008). Data from Van Strien et al. (1995) indicate that emotional eating is associated with emotional problems, such as depression, suicidal ideas, anxiety, problems with relationship, and sex, which might indicate that emotional eaters have a general susceptibility for negative affect. Body dissatisfaction, which has previously been associated with negative affect, might impede emotional eaters to cope with the negative feelings (e.g., negative self-conscious emotions predisposition such as shame and guilt-proneness) and, consequently, increase the frequency of emotional eating (Van Strien et al., 2005; Cavalera et al., 2016). In the context of the COVID-19 pandemic, the relation found between body dissatisfaction and emotional eating might be explained by the increase in negative affects brought by COVID-19-related stress, anxiety, and depression. Indeed, stress, anxiety, and depression have exacerbated in populations mainly by its new and unexplored characteristics and by the uncertainty about the future that accompanies it (Gallagher et al., 2020; Kujawa et al., 2020; Salari et al., 2020). Time spent on social media also increased significantly during the COVID-19 pandemic, probably because of physical distancing (Cellini et al., 2020). Researchers report a significant increase in the frequency of use of appearance-centered social network sites, especially in women (Vall-Roqué et al., 2021), which have previously been associated with coping difficulties, low self-esteem, and body dissatisfaction (Kostanski and Gullone, 1998; Anderson et al., 2011; Holland and Tiggemann, 2016; Cohen et al., 2017). Thus, stress, anxiety, and time spent on social networks may have diminished coping skills to manage threats against messages conveyed by social media on body image and, consequently, might increase body dissatisfaction, which is known to lead individuals to eat as a way to calm or to reward themselves (Geller et al., 2019; Al-Musharaf, 2020).

Body dissatisfaction as well as weight and shape concerns were not significantly related to self-rated changes in the frequency of overeating among women of our sample. These results go against what was expected. Indeed, the relationship between overeating and negative body image has been shown to be significant in the literature (e.g., Ahrberg et al., 2011; Holmes et al., 2015; Linardon et al., 2019). Our results suggest that the relationship between negative body image and overeating episodes should be further explored. We could hypothesize that in our sample, overeating episodes would not be predicted by negative body image, but by emotional distress caused by the COVID-19 pandemic, as suggested by the dual-pathway model proposed by Stice (1994, 2001) to explain bulimic behavior. Specifically, Stice proposed that body dissatisfaction and bulimic behavior could be linked though the pathway of negative emotions. Overeating could represent an escape from experiencing negative emotions (Heatherton and Baumeister, 1991; Van Strien et al., 2005; Goldfield et al., 2008). Van Strien et al. (2005) found support for the pathway of negative affect but they also propose an extended pathway model. The relationship between negative affect and overeating could be explained by emotional eating and a lack of interoceptive awareness. More precisely, the authors found a strong relation between emotional eating and overeating/binge eating in a clinical sample, and both interoceptive awareness and emotional eating as intermediating variables between negative affect and overeating/binge eating. Van Strien et al. (2005) did not find the same pathway in their community sample of female students. Specifically, the authors found a weaker, although still significant, relation between emotional eating and overeating/binge eating. The lack of interoceptive awareness was strongly associated with negative affect but did not totally explain the relation between negative affect and emotional eating. Future studies should include instruments to measure negative affect (e.g., shame and guilt) and interoceptive awareness to shed light on the possible predictors of overeating episodes.

The findings of this study should be interpreted in light of some limitations. First, this study included self-reported measures in which participants were asked to indicate perceived changes in frequency of eating habits. It is possible that women may not be reporting their eating behaviors accurately. Second, since the study relies on a convenience sample, results may not be generalizable to younger and/or older women and to women from different countries. Furthermore, our findings cannot be generalized to men and individuals with a current or history of eating disorders as they were excluded from this study. Third, body dissatisfaction was only measured only with visual depictive body size estimation tasks using the *e*LoriCorps-IBRS v 1.0 (Monthuy-Blanc et al., 2020). It could be interesting to measure body dissatisfaction with another type of instrument, such as a questionnaire. Indeed, it has been suggested that different instruments could assess different dimensions of body image-related construct (Moelbert et al., 2017). Finally, this is a cross-sectional study that does not allow us to interpret associations between variables as reflecting causal relations. Longitudinal studies could allow examining the long-term contribution of body image-related variables on women’s problematic eating behaviors.

The results of this study can have important preventive and therapeutic implications. As we are entering the third year of the pandemic, it is critical to understand how people have been affected so far, and how to prepare to mitigate the impact of a prolonged pandemic crisis. A possible strategy could be the implementation of cognitive and behavioral body image interventions combined with intuitive eating education programs. Cognitive and behavioral body image interventions could help ameliorate negative body image by implementing and adopting daily life techniques that can alter the dysfunctional affective and cognitive dimensions of body image and promoting a more positive body image (e.g., Butters and Cash, 1987; Jarry and Berardi, 2004; Jarry and Ip, 2005; Cash, 2008). Cognitive-behavioral models consider negative body image (e.g., shape and weight concerns and body dissatisfaction) as the core of disordered eating symptomatology (Fairburn et al., 2003). However, before directly targeting negative body image (during, for instance, cognitive-behavior interventions), it is suggested to indirectly reduce its influence by targeting other disordered eating symptoms, such as restrictive eating, overeating episodes, and emotional eating (Fairburn, 2008; Linardon and Mitchell, 2017). Intuitive eating can be defined as having a strong connection with one’s own internal signals of hunger and satiety cues, and consequently eating in response to these signals (Tylka, 2006; Messer et al., 2021). Increasing scientific evidence is pointing to the potential positive role of intuitive eating as a protective factor against several eating disorder symptoms such as disordered eating, binge eating, restrictive eating, and unhealthy weight control behaviors (Dockendorff et al., 2012; Hazzard et al., 2020; Christoph et al., 2021; Linardon et al., 2021; Messer et al., 2021; Sanlier et al., 2021). Intuitive eating appears to be associated also to lower body image concerns, higher positive body image, body appreciation, self-esteem, self-compassion, self-determination, body empowerment, and wellbeing (Dockendorff et al., 2012; Tylka and Wood-Barcalow, 2015; Bruce and Ricciardelli, 2016; Messer et al., 2021; Sanlier et al., 2021).

## Conclusion

The effect of the pandemic on body image and eating behaviors remains largely understudied. This paper highlights the existence of a positive relation between negative body image and increased frequency of disordered eating behaviors in a community sample of women during the COVID-19 crisis. These results should be taken into consideration, as they shed light on a dangerous pattern of phenomena in a non-clinical sample of women. They seem of critical importance as they represent the key risk factors for the development of eating disorders. Our hope is that future studies would follow and that we would be able to reach a better understanding about how people were affected by this global crisis to promptly react with the development and implementation of prevention programs.

## Data Availability Statement

The data for this study is available upon request addressed directly to the Research Ethics Boards (comite.ethique@uqo.ca). The dataset is not publicly available due to privacy and ethical restrictions.

## Ethics Statement

The studies involving human participants were reviewed and approved by ethical committee of Université du Québec en Outaouais (Quebec, Canada) and of Université du Québec à Trois-Rivières (Quebec, Canada). The patients/participants provided their written informed consent to participate in this study.

## Author Contributions

GC, AP, JM-B, and SB created and conceptualized the study. GC, AP, and MO collected the data. GC, AP, and SB analyzed and interpreted the data. GC and AP wrote the first draft. GC, AP, JM-B, MO, and SB critically revised the manuscript and provided constructive comments. All authors contributed to the article and approved the submitted version.

## Funding

This work was supported by a postdoctoral grant awarded to the first author (GC) by the Fond de Recherche du Québec— Santé (FRQS; 289006); the Canada research Chair in clinical cyberpsychology (950-210762)awarded to SB; and the RBC Royal Bank, the Lemaire family, Fond. UQTR-RBC Banque Royale-PI-Loricorps, 2017–2021, by the Fonds de Recherche du Québec- Société et culture, FRQSC-2014-NP-17646, 2013–2020, by the Social Sciences and Humanities Research Council of Canada, 2018–2023 (CRSH-SAVOIR-430-2018-00970–PIE-IC), and the settlement fund of the Centre de Recherche de l’Institut Universitaire en Santé Mentale de Montréal, awarded to JM-B.

## Conflict of Interest

The authors declare that the research was conducted in the absence of any commercial or financial relationships that could be construed as a potential conflict of interest.

## Publisher’s Note

All claims expressed in this article are solely those of the authors and do not necessarily represent those of their affiliated organizations, or those of the publisher, the editors and the reviewers. Any product that may be evaluated in this article, or claim that may be made by its manufacturer, is not guaranteed or endorsed by the publisher.
