# Supplementary material for: The Relationship Between Women’s Negative Body Image and Disordered Eating Behaviors During the COVID-19 Pandemic: A Cross-Sectional Study
Source: Front Psychol. 2022 Mar 24;13:856933. doi: 10.3389/fpsyg.2022.856933 (PMC8987766; doi:10.3389/fpsyg.2022.856933)
Supplement: Supplementary file 1 [file Data_Sheet_1.docx]

**Appendix A - Supplementary material**

**Figure 1.** Skin color scale used in this study


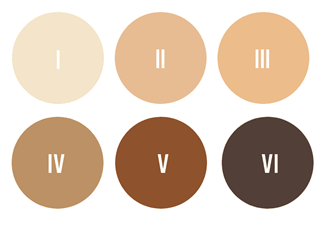


*Note.* I= very light skin color; II= light skin color; III= intermediate skin color; IV= tan skin color; V= brown skin color; VI= dark skin color

**Table 1.** One sample t tests for self-rated changes in frequency of disordered eating behaviors based on retrospective self-report (n=152)

|  | Restrictive eating | Overeating | Emotional eating |
| --- | --- | --- | --- |
|  | n (%) | n (%) | n (%) |
| 1 = Much less than before | 5 (3.3) | 5 (3.3) | 2 (1.3) |
| 2 = Less than before | 6 (3.9) | 3 (2.0) | 1 (0.7) |
| 3= A little less than before | 10 (6.6) | 4 (2.6) | 10 (6.6) |
| 4= No change from before | 80 (52.6) | 92 (60.5) | 66 (43.4) |
| 5= A little more than before | 33 (21.7) | 37 (24.3) | 53 (34.9) |
| 6 = More than before | 14 (9.2) | 7 (4.6) | 11 (7.2) |
| 7 = Much more than before | 4 (2.6) | 4 (2.6) | 9 (5.9) |
|  | *M* (*SD*) | *M* (*SD*) | *M* (*SD*) |
|  | 4.24 (1.14) | 4.25 (1.01) | 4.55 (1.04) |
| t(151) | 2.57 | 3.07 | 6.55 |
| *d* | .21 | .25 | .53 |
| *p*-value | .011 | .003 | < .001 |

**Table 2.** Pearson’ correlations (n= 152)

| Variable | 1 | 2 | 3 | 4 | 5 | 6 |
| --- | --- | --- | --- | --- | --- | --- |
| 1. Restrictive eating | -- | -.03 | -.01 | .27^**^ | .24^**^ | .08 |
| 2. Overeating | -.03 | -- | .45^***^ | -.05 | .01 | .20 |
| 3. Emotional eating | -.01 | .45^***^ | -- | .00 | .00 | .25^*^ |
| 4. Weight concerns ^a^ | .27^**^ | -.05 | .00 | -- | .89^***^ | .50^***^ |
| 5. Shape concerns ^b^ | .24^**^ | .01 | .00 | .89^***^ | -- | .48^***^ |
| 6. Body dissatisfaction ^c^ | .08 | .20 | .25^*^ | .50^***^ | .48^***^ | -- |

*Note*. ^*^*p* < .05; ^**^*p* < .01; ^***^*p* < .001;

N = 152, except for ^a^ n= 130, ^b^ n= 131, ^c^ n= 97
